# Enhancing memory capacity by experimentally slowing theta frequency oscillations using combined EEG-tACS

**DOI:** 10.1038/s41598-022-18665-z

**Published:** 2022-08-20

**Authors:** Tuba Aktürk, Tom A. de Graaf, Bahar Güntekin, Lütfü Hanoğlu, Alexander T. Sack

**Affiliations:** 1 Program of Electroneurophysiology, Vocational School, Istanbul Medipol University, Istanbul, Turkey; 2 Section Brain Stimulation and Cognition, Department of Cognitive Neuroscience, Faculty of Psychology and Neuroscience, Maastricht University, Maastricht, The Netherlands; 3 Department of Neurology, School of Medicine, Istanbul Medipol University, Istanbul, Turkey; 4 Department of Biophysics, School of Medicine, Istanbul Medipol University, Istanbul, Turkey; 5 Research Institute for Health Sciences and Technologies (SABITA), Istanbul Medipol University, Istanbul, Turkey

**Keywords:** Cognitive neuroscience, Learning and memory

## Abstract

The coupling of gamma oscillation (~ 40+ Hz) amplitude to the phase of ongoing theta (~ 6 Hz) oscillations has been proposed to be directly relevant for memory performance. Current theories suggest that memory capacity scales with number of gamma cycles that can be fitted into the preferred phase of a theta cycle. Following this logic, transcranial alternating current stimulation (tACS) may be used to adjust theta cycles (increasing/decreasing theta frequency) to decrease or increase memory performance during stimulation. Here, we used individualized EEG-informed theta tACS to (1) experimentally “slow down” individual theta frequency (ITF), (2) evaluate cognitive *after effects* on a battery of memory and learning tasks, and (3) link the cognitive performance changes to tACS-induced effects on theta-band oscillations as measured by post EEG. We found frequency- and task-specific tACS after effects demonstrating a specific enhancement in memory capacity. This tACS-induced cognitive enhancement was specific to the visual memory task performed immediately after tACS offset, and specific to the ITF-1 Hz (slowing) stimulation condition and thus following a protocol specifically designed to slow down theta frequency to enhance memory capacity. Follow-up correlation analyses in this group linked the enhanced memory performance to increased left frontal-parietal theta-band connectivity. Interestingly, resting-state theta power immediately after tACS offset revealed a theta power increase not for the ITF-1 Hz group, but only for the ITF group where the tACS frequency was ‘optimal’ for entrainment. These results suggest that while individually calibrated tACS at peak frequency maximally modulates resting-state oscillatory power, tACS stimulation slightly below this optimal peak theta frequency is better suited to enhance memory capacity performance. Importantly, our results further suggest that such cognitive enhancement effects can last beyond the period of stimulation and are linked to increased network connectivity, opening the door towards more clinical and applied relevance of using tACS in cognitive rehabilitation and/or neurocognitive enhancement.

## Introduction

Brain oscillations contribute to different cognitive functions according to different characteristics of the oscillatory signal itself (amplitude, frequency, phase, coherence, power, cross-frequency couplings) as well as their specific topologies^1–4^. For instance, theta oscillations (~ 6 Hz) measured from fronto-parietal network regions have been associated with memory processes. It is known that cortical theta oscillations reflect communication with the hippocampus, which has been proposed as a driver of memory functions^4–13^. Gamma oscillations (~ 40+ Hz), depending on the cortical region, have also been associated with several (sub) cognitive processes that contribute to memory, such as conscious perception^14^, selective processing of information^15,16^, and active preservation of memory content^17^.

Memory processes are part of the foundation of human cognition, being closely related to other core functions such as attention or executive control^18–20^. Therefore, it is unsurprising to see interaction of theta oscillatory characteristics with oscillations in other frequency bands, associated with other high-level cognitive functions. In this sense, theta-gamma coupling appears to be an ideal mechanism for linking representations or operations from different neurocognitive sources into a cohesive mental representation. Lisman and Idiart^21^, for example, proposed that the number of gamma cycles that fit into one theta cycle determines the limits of memory capacity^22^. In other words, a longer theta cycle implies higher memory capacity by accommodating more gamma cycles. Several correlational studies provided evidence to support this suggested relationship between memory performance and theta-gamma cross-frequency coupling^7,8,21–25^.

Transcranial alternating current stimulation (tACS) has been used to modulate and entrain endogenous brain oscillations, allowing causal investigation of hypothesized roles of oscillations in human cognition. TACS delivers low-intensity electrical current between two electrodes, alternating in polarity at predetermined frequencies associated with targeted cognitive processes. Since a slow oscillating theta can accommodate more cycles of fast gamma, previous studies evaluated whether tACS can be used to increase memory capacity by decreasing the frequency of theta^26–28^.

In most of these studies, tACS was used to entrain brain oscillations and enhance/modulate cognitive functions *during* the tACS application (i.e., online effects). Although interesting and promising, such online tACS effects are potentially confounded by sensory entrainment effects (indirect entrainment) caused by the sensation of the tACS application on the skin. In contrast, a successful demonstration of modulatory effects beyond the period of stimulation (*after effects*) would not only be less confounded by these sensory stimulation side effects, but also paramount for gaining new insights into the oscillatory processes underpinning possible tACS-induced changes. Finally, offline tACS memory effects would also open the door towards a more clinical and applied relevance of using tACS in cognitive rehabilitation and/or neurocognitive enhancement.

The oscillations entrained during rhythmic stimulation have been shown to be sustained for only a few oscillatory cycles after the stimulation is turned off^29–32^. These so-called “entrainment-echoes” are short-lived post-rhythmic resonances that are locked to the entraining field^31^. Most of the offline effects on neural oscillations, on the other hand, can be seen over considerably longer time periods (e.g.,^33–35^) and may thus not represent the continuance of online entrainment^32^. There are some promising studies on the after effects of “theta” tACS on both brain activity and behavior^36–39^, although most of these studies did not show direct evidence of the behavioral after effect of theta tACS^26,40^. These findings regarding the after effects of tACS are mostly discussed in the context of the “spike timing dependent plasticity” mechanism^32^. Theta-frequency after effects on EEG-correlates of tACS-induced behavioral changes are less established in the literature, except for some inconsistent evidence (for review, see Veniero et al.^32^). Therefore, investigating the behavioral and physiological after effects of theta tACS as presented here can also be considered important to start better understanding possible plasticity-related changes of tACS that go beyond the short-lived entrainment effects.

In the current study, we assessed potential after effects of a tACS protocol aiming to “slow down” the individual theta frequency (ITF), with the goal of enhancing memory performance capacity beyond the period of stimulation. Furthermore, we evaluated after effects on resting state EEG and memory task related EEG, with a focus on theta-band oscillations. To this end, we administered tACS at 1 Hz slower than ITF (ITF-1), to experimentally slow down theta frequency and thereby enhance memory performance^26,28,41^. In a between-subject design, participants were randomly assigned to three stimulation conditions: (1) tACS at ITF (no frequency change, but stimulating at individually calibrated optimal peak frequency for most effective theta entrainment), (2) tACS at ITF-1 (for slowing down ITF, changing frequency but no or less entrainment), and SHAM tACS (no frequency change, no entrainment). TACS was applied over left frontoparietal network, as part of the neural networks underlying memory and learning functions^6,27,39,42–45^.

In light of the inconsistent behavioral findings of studies using theta tACS across different task types^32,40,46,47^, it is often assumed that the theta tACS effect may vary for different tasks and/or task modalities. Therefore, here, different tasks/tests (e.g., both visual and auditory memory tasks) were applied as an exploratory approach to test task-specificity of tACS after effects on behavioral performance. Based on our a priori hypothesis-driven main research question, tACS-related behavioral after effects were expected especially in memory-related tasks and for the ITF-1 group, due to the here induced “theta slowing” as compared to the ITF and sham conditions. In contrast, and in accordance with previous findings, EEG power spectrum changes were expected exclusively in the ITF group (entrainment) since only in this condition, tACS was administered at the individual peak frequency (e.g.^33,35,48,49^). Since we aimed to study after effects of theta tACS, stimulation was applied at rest (for 20 min), and memory/learning tasks and EEG measurements were obtained both before and after the tACS interventions.

## Results

In this between-subject tACS-EEG experiment, different groups of participants performed various memory tasks before and after individual theta-frequency tACS (ITF), slowed theta tACS (ITF-1 Hz), and SHAM tACS. Here, after an analysis of participants’ reports on side effects and blinding success, we evaluate tACS after effects on memory performance and on EEG (time)-frequency activity respectively.

### Blinding success and side effects

Before the experiment, participants were asked to rate their belief in the general effectiveness of the tACS application (between 0 and 10); greater values indicate greater belief in the efficacy of tACS. The belief in the effect of the tACS application didn’t differ across groups (X^2^(2) = 1.45, p = 0.484) (ITF group: mean/sd: 5.20 ± 1.42, ITF-1 group: mean/sd: 5.62 ± 1.63, Sham group: mean/sd: 5.40 ± 2.41). At the end of their session, participants were asked whether they thought the tACS they received was real or placebo. There were descriptive, but not statistically significant, differences between groups (X^2^(4, N = 46) = 6.33, p = 0.176) (ITF group: Real/Placebo/Don’t Know: 9/1/5, ITF-1 group: Real/Placebo/Don’t Know: 10/0/6, Sham group: Real/Placebo/Don’t Know: 6/4/5). After the (sham) tACS session, participants filled out a side effect questionnaire including 7 items (e.g., itching, fatigue, etc.) (see “Methods”). Scores were summed to yield an individual ‘side effect score’ that did significantly differ between groups (X^2^(2) = 7.02, p = 0.030) (ITF group: mean/sd: 5.73 ± 2.83, ITF-1 group: mean/sd: 4.56 ± 2.90, Sham group: mean/sd: 2.80 ± 2.43), with fewer side effects reported by the sham group as compared to the ITF group (pairwise comparison, p = 0.033). In sum, all groups were essentially identical in a priori ‘belief’ about tACS efficacy, not significantly different in their estimations of real vs placebo treatment, somewhat different in terms of reported side effects. We conclude that, in combination with the fact that we only measured behavior and EEG before and after tACS and not during tACS, these results raise no concerns about the validity of our between-subject model.

### Behavioral results

Participants performed two memory tasks before, and again after, their 20-min (sham) tACS protocol; a visual memory task (VM), an auditory memory task (AM) during the EEG recording. The tasks were always administered in that order, which means that the visual memory task was always started approximately 6 min after offset of tACS (after the resting state EEG), and the auditory memory task always started approximately 14 min after offset of tACS (after the visual memory task). We performed mixed analyses of variance (ANOVA) with factors time (pre-tACS and post-tACS) and group (ITF tACS, ITF-1 Hz tACS, sham tACS) separately per task.

**Figure 1:**
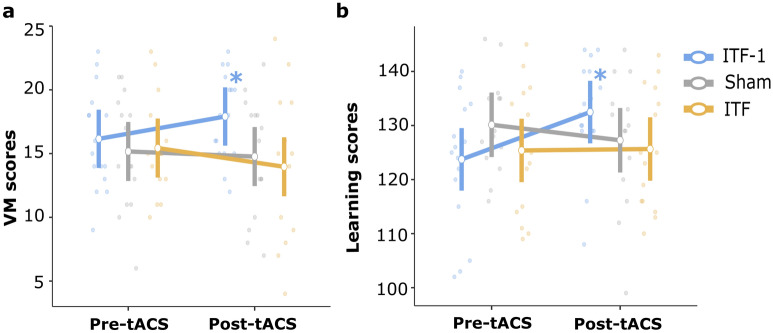
The significant behavioral results. (**a**) The mean values of the VM scores at pre- and post-tACS across the groups. The time*group interaction was significant (p = 0.016). The ITF-1 group had an increased VM score after the tACS. (**b**) The mean values of the Learning scores at pre- and post-tACS across the groups. The time*group interaction was significant (p = 0.011). ITF-1 group had an increased learning score after the tACS. See Supplementary Fig. 1 for additional representation. VM, visual memory; tACS, transcranial alternating current stimulation; ITF, individual theta frequency. The vertical bars denote 0.95 confidence intervals. Dots represent the observed scores. Asterisks indicate line reflects a significant change over time.

The time*group interaction was significant for the VM task (F(2, 43) = 4.54, p = 0.016, and η_p_^2^ = 0.174) but not for the AM task (F(2, 43) = 1.26, p = 0.293, and η_p_^2^ = 0.055). Follow-up pairwise comparisons showed that specifically in the ITF-1 group, where the protocol aimed to slow the theta frequency in order to enhance memory capacity, VM performance was enhanced after tACS (mean VM score pre-tACS: 16.1 ± 3.9 and post-tACS: 17.9 ± 3.4), (uncorrected t(43.0) = − 2.306, p = 0.026, d = 0.48), with no significant change in the ITF (pre-tACS: 15.4 ± 3.9, post-tACS: 13.9 ± 5.6) (uncorrected t(43.0) = 1.871, p = 0.068, d = − 0.31) and Sham (pre-tACS: 15.1 ± 4.5, post-tACS: 14.7 ± 4.7) (uncorrected t(43.0) = 0.510, p = 0.612, d = − 0.09) groups (Fig. 1a). See Supplementary Table 1 for means and SDs of all task scores across the groups and times (pre-post).

Participants were evaluated with the neuropsychological battery before, and after, their tACS protocol; an Oktem verbal memory test (OVMT), Rey complex figure test (RCFT), digit span test, and letter-number sequencing test. We performed mixed ANOVA with factors time (pre-tACS and post-tACS) and group (ITF tACS, ITF-1 Hz tACS, sham tACS) separately per outcome measure. In the analyses of the OVMT, one subject was removed since the subject was familiar with the test.

The time*group interaction was significant only for the OVMT learning sub-score (F(2, 42) = 5.02, p = 0.011, and η_p_^2^ = 0.193). Post hoc pairwise comparisons show that ITF-1 group had an increased learning score after the tACS (mean learning score of the pre-tACS: 124 ± 12.2 and post-tACS: 133 ± 9.79) (uncorrected t(42.0) = − 3.343, p = 0.002, d = 0.81) while there was no change in the ITF (mean learning score of the pre-tACS: 125 ± 11.8 and post-tACS: 126 ± 10.9) (uncorrected t(42.0) = − 0.099, p = 0.922, d = 0.09) and Sham (mean learning score of the pre-tACS: 129 ± 9.65 and post-tACS: 127 ± 12.2) (uncorrected t(42.0) = − 3.343, p = 0.313, d = − 0.18) groups (Fig. 1b). Short-term memory (F(2, 42) = 0.76, p = 0.472, and η_p_^2^ = 0.032) and long-term memory (F(2, 42) = 2.92, p = 0.065, and η_p_^2^ = 0.122) sub-scores of the OVMT were not different between tACS groups. There were no time*group interactions for the other measures: short-term memory (F(2, 43) = 0.63, p = 0.539, and η_p_^2^ = 0.028) or long-term memory (F(2, 43) = 0.79, p = 0.458, and η_p_^2^ = 0.036) sub-scores of the RCFT, or digit span forward (F(2, 43) = 0.55, p = 0.581, and η_p_^2^ = 0.025) and letter-number sequencing (F(2, 43) = 0.212, p = 0.626, and η_p_^2^ = 0.022) tests. See Supplementary Table 2 for means and SDs of all test scores across the groups and times (pre-post).

### EEG results

We measured EEG at rest, before tACS and immediately following tACS, as well as during the various behavioral tasks. Of those, we here focused on and report EEG time–frequency activity in the theta range during the visual and auditory memory tasks that immediately followed the tACS and subsequent resting state EEG.

#### Resting EEG

In the analyses, we only used the 3-min eyes-open resting EEG recording since we anticipated less obscured theta activation by strong alpha activation. We performed Fast Fourier Transform to create power spectra that allowed us to extract (1) peak frequency in the theta range, and (2) power in a theta window + /− 1.5 Hz around that individual stimulation frequency.

Please, see supplementary material for the results of the first set ANOVAs which were included location and hemispheres as the within-subjects factors.

**Figure 2:**
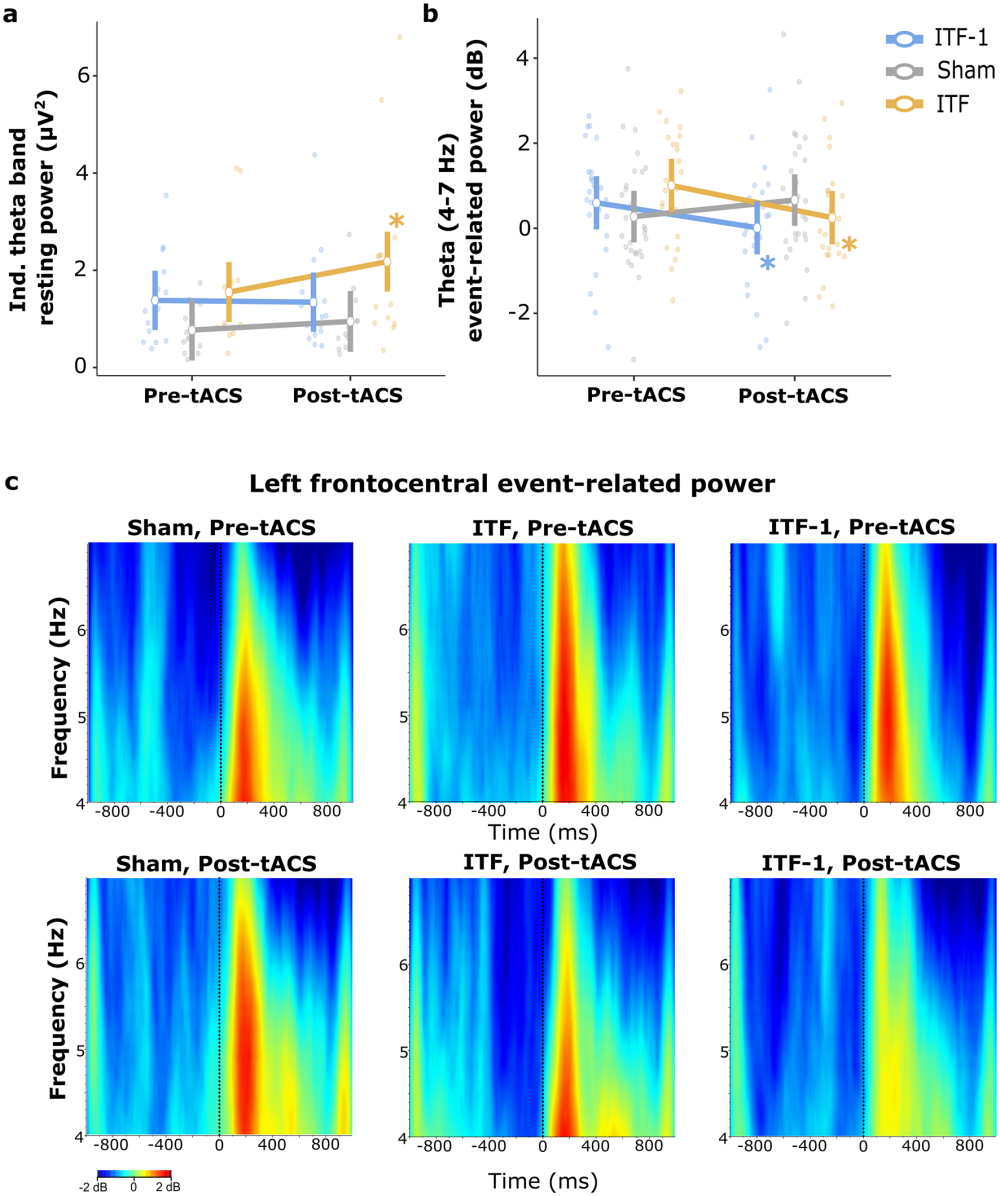
The significant results of the after effect of the tACS on EEG data. (**a**) The time*group interaction was significant (p = 0.016): ITF group had increased resting theta power after tACS while there were no tACS effects on the other groups. See Supplementary Fig. 2 for additional representation. (**b**) The time*group interaction was significant (p = 0.01) for VM task. The tACS groups had decreased event-related theta power after tACS while no such difference was observed in the sham group. See Supplementary Fig. 3 for additional representation. (**c**) The grand average figures of event-related power analysis (4–7 Hz) in time–frequency domain in response to items in the VM task. The left frontocentral area was presented for each group in the figure. tACS groups had decreased event-related theta power after theta-frequency tACS (for ITF-1 p = 0.045, for ITF P = 0.011), not observed in the sham group (p = 0.145). The X-axis represents time, and the Y-axis represents frequency; the point at which the stimulus arrives is marked as a zero point on the X-axis. tACS, transcranial alternating current stimulation; ITF, individual theta frequency. The vertical bars denote 0.95 confidence intervals. Dots represent the observed scores. Asterisks indicate line reflects a significant change over time.

In a second ANOVA, more focused set of statistical analyses, we removed location and hemisphere factors to analyze specifically the left frontocentral EEG around the site of tACS stimulation. Here, four outlier subjects (see “Methods”) were removed (two from sham, one from the ITF, and one from the ITF-1 group). Now the ANOVA revealed that resting-state theta-band power values increased from pre-tACS to post-tACS (main effect of ‘time’: F(1, 39) = 7.74, p = 0.008, and η_p_^2^ = 0.166), and that this effect depended on group (time*group interaction: F(2, 39) = 4.62, p = 0.016, and η_p_^2^ = 0.192). Follow-up pairwise comparisons showed that specifically the ITF group had increased theta power after tACS (p < 0.001) while we saw no tACS (‘time’) effects for the other groups (for ITF-1 p = 0.816, for sham p = 0.288) (Fig. 2a). There were no aftereffects of tACS on the maximum peak frequency (time*group interaction: F(2, 43) = 1.38, p = 0.263, and η_p_^2^ = 0.06). It is interesting that, when it comes to finding a local increase in resting state theta power, this was specifically (and highly significantly) obtained in the group where tACS explicitly targeted the individual peak frequency. At the same time, note in Fig. 2a the substantial inter-individual variability.

We performed resting coherence analysis over the FFT in the theta frequency range between F3 and P3 electrodes where we positioned the tACS electrodes. We again employed a mixed time (pre-tACS, post-tACS) by group (ITF, ITF-1, Sham) ANOVA, removing two outlier participants (one from the ITF and one from the ITF-1 group). While this analysis revealed a strong main effect of time (F(1, 41) = 17.59, p = 0.0001, and η_p_^2^ = 0.300), with higher resting theta coherence after tACS, there was no interaction with group (F(2, 41) = 1.13, p = 0.333, and η_p_^2^ = 0.052).

#### Event-related EEG

We used complex Morlet Wavelet Transform (WT) to extract event-related theta band (4–7 Hz) activity in the time–frequency domain, time-locked to presentation of visual/auditory items in the visual/auditory memory tasks.

As in the resting EEG, the results of the first set of ANOVAs which include location and hemispheres as within-subjects factors were presented in the supplementary material of the current paper.

In the second set of statistical analyses, we removed factors location and hemisphere and focused on left frontocentral EEG, around the site of tACS stimulation. The auditory task ANOVA revealed no after effects of tACS on event-related theta power (time*group interaction: F(2, 29) = 0.25, p = 0.781, and η_p_^2^ = 0.017). However, in the analogous ANOVA on event-related theta-band power during the visual memory task, where we reported a behavioral time*group interaction, we found a significant time*group interaction (F(2, 35) = 5.32, p = 0.01, and η_p_^2^ = 0.233). Follow-up pairwise comparisons showed that both tACS groups had decreased event-related theta power after theta-frequency tACS (for ITF-1; p = 0.045, for ITF; p = 0.011), not observed in the sham group (p = 0.145) (Fig. 2b, c).

The event-related power-based connectivity analysis in the time–frequency domain used in the current study measures similarity between F3 and P3 electrodes where tACS stimulation electrodes were placed on. The Phase Locking Value (PLV) used in the current study is a phase-based connectivity metric in the time–frequency domain, and it indicates phase-lag consistency of specified channels (F3 and P3). We performed mixed-design ANOVAs with factors of time (pre-tACS and post-tACS), item encoding (remembered, forgotten), and group (ITF tACS, ITF-1 Hz tACS, sham tACS).

In the statistical analyses of event-related power-based connectivity, two outlier subjects were removed (one subject from the VM task and one from the AM task). There were no significant after effects of tACS on VM (time*group interaction: F(2, 34) = 1.17, p = 0.322, and η_p_^2^ = 0.065) and AM (time*group interaction: F(2, 28) = 0.075, p = 0.928, and η_p_^2^ = 0.005) tasks.

In the statistical analyses of PLV, no significant after effects of tACS on VM (time*group interaction: F(2, 35) = 2.19, p = 0.127, and η_p_^2^ = 0.111) or AM (time*group interaction: F(2, 29) = 0.308, p = 0.738, and η_p_^2^ = 0.021) tasks were shown.

### EEG-behavior correlations

Since we observed tACS after effects specifically in the visual memory (VM) task both in behavioral and EEG data, we focused on the VM task in statistical follow-up analyses of EEG-behavior interactions. Here, we removed the same outliers as in the EEG analyses above.

**Figure 3:**
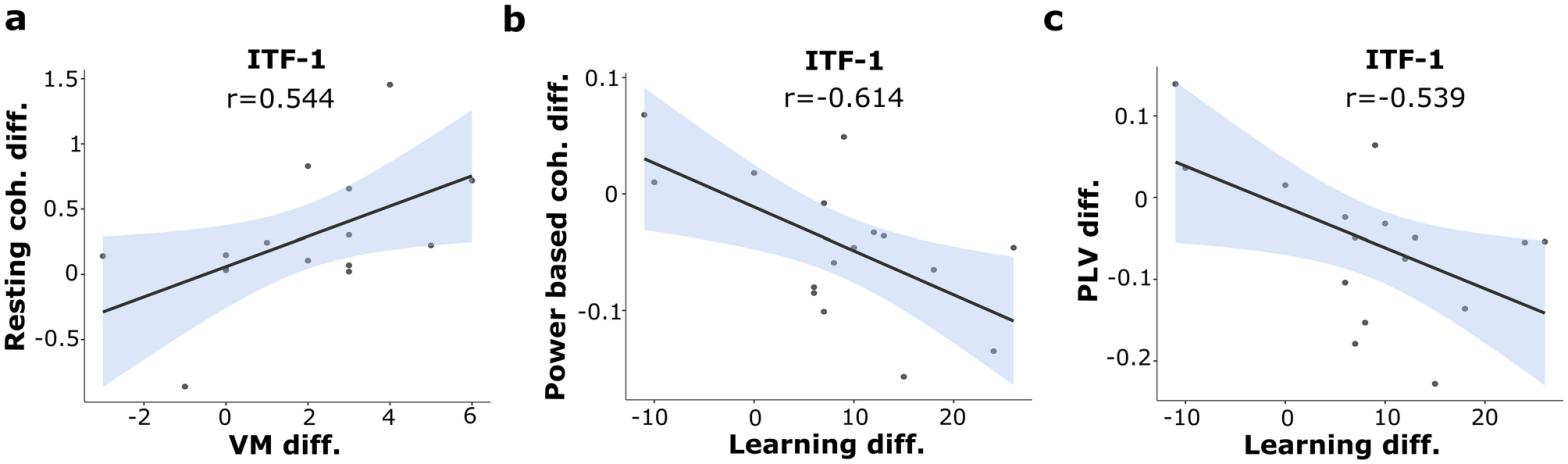
The scatter plots of the significant results in the EEG-behavior correlation analyses. (**a**) The subjects in the ITF-1 group with increased left frontal-parietal resting theta coherence after the tACS had the higher scores in the VM task (r = 0.544, p = 0.036). (**b**) The subjects in the ITF-1 group with decreased left frontal-parietal event-related power-based theta connectivity after the tACS had the higher learning scores (r = − 0.614, p = 0.011). (**c**) The subjects in the ITF-1 group with decreased left frontal-parietal PLV after the tACS had the higher learning scores (r = − 0.539, p = 0.031). Other groups' scatter plots were presented in the Supplementary Fig. 4. Coh., coherence; Diff., difference score; PLV, phase locking value; VM, visual memory; ITF, individual theta frequency. The shaded area denotes the standard error. Dots represent the observed scores.

There were no significant correlations between changes in behavioral data (difference scores for learning and VM: post tACS minus pre tACS values) and changes in left frontocentral event-related theta power, resting-state theta power, or resting state maximum peak frequency analysis (difference score: post tACS minus pre tACS values) for any group (p > 0.05 for all). However, there was a positive correlation between tACS effects on left frontal-parietal *resting-state* theta coherence, and tACS effects on the VM task, specifically in the ITF-1 group (r = 0.544, p = 0.036; ITF group p = 0.559; Sham group p = 0.303) (Fig. 3a). There was no such correlation with learning difference scores (p > 0.05 for all). Instead, we observed a strong negative correlation between tACS effects on left frontal-parietal *event-related* power-based theta connectivity and learning scores (r = − 0.614, p = 0.011) (Fig. 3b), again specifically for the ITF-1 group (ITF group p = 0.667; Sham group p = 0.431). This time, no such correlation with VM scores. Finally, there was also a negative correlation in specifically the ITF-1 group between tACS effects on specifically learning scores (not VM) and left frontal-parietal PLV (ITF-1 group r = − 0.539, p = 0.031; ITF group p = 0.897; Sham group p = 0.737) (Fig. 3c).

In sum, in the search for EEG-correlates of tACS induced behavioral changes (after effects), coherence measures (between left frontal and left parietal electrodes) were found significant in the current dataset. Future studies might further explore these correlations between tACS effects on visual memory scores and on resting-state theta coherence, as well as tACS effects on learning scores in relation to event-related coherence (power-based and PLV). We consider these results exploratory, to be interpreted only with caution, given the number of statistical tests and lack of corresponding correction of statistical threshold.

Additionally, to control whether pre-tACS EEG activity affected the after effect of the tACS on behavior, ANCOVAs for repeated measures were performed for the VM and total learning scores, separately, with the between-subjects factor of the group (ITF, ITF-1, Sham), within-subjects factors of time (pre-tACS, post-tACS), and the “pre-tACS EEG data” as the covariate. As the “pre-tACS EEG data”, the same EEG measures used in the correlation analysis were employed as covariates in the ANCOVAs, however, not the difference scores as in the correlations, but the pre-tACS EEG values. These covariates were: left frontocentral theta values of the event-related power, resting power, resting maximum peak frequency, F3-P3 theta connectivity values of the event-related power- and phase-based connectivity, as well as resting coherence. In the ANCOVA results, the behavioral after effect of the tACS on the learning and VM scores in the ITF-1 group remained significant, and the results showed pre-tACS EEG data have no influence on the behavioral after effect of tACS. See supplementary material for detailed results of ANCOVAs.

## Discussion

Here we investigated cognitive and neurophysiological after effects of individualized EEG-informed theta-frequency tACS designed to either entrain or slow down the natural endogenous theta oscillations in order to enhance memory capacity. The goal was to experimentally ‘slow’ theta oscillations by tACS stimulation at 1 Hertz below the individual peak theta frequency (ITF-1, slowing), and to compare results to peak-theta-frequency tACS (ITF, entraining) and to placebo tACS (SHAM). Theoretical models^21,22^ predict that lower-frequency theta oscillations would enable enhanced memory performance through the facilitation of more gamma-range activity at a preferred theta phase. Simply put, these theoretical concepts suggest that at slower theta frequencies more gamma cycles can be fitted into the preferred phase of a theta cycle, a form of theta-gamma cross frequency coupling that has been linked to memory capacity. Here, we tested for the first time whether i) such effects can be observed after the offset of tACS (i.e., in the form of after-effects), ii) such effects are indeed frequency specific, and iii) what neurophysiological correlates can be associated to such tACS induced cognitive enhancements. TACS was applied at rest, since we were explicitly interested in after-effects with EEG being recorded before and after tACS during resting-state, visual memory (VM), and auditory memory (AM) task execution (during the encoding phase). Additionally, before and after tACS, a battery of behavioral and neuropsychological tests was used to evaluate attention, learning, and memory performances.

The main results of the study can be summarized in three domains: behavior, EEG-brain oscillations, and behavior-EEG interactions. In terms of *behavior*, we found after-effects of tACS on memory performance in terms of enhanced visual memory capacity and higher learning scores in specifically the ITF-1 (slowing) condition. In the *EEG* data, we observed decreased frontocentral event-related theta power in both tACS conditions for the VM task, while frontocentral resting state theta power increased in the ITF group following the tACS session. *Behavior-EEG interaction* analyses suggested that subjects in the ITF-1 group with enhanced left fronto-parietal resting theta connectivity after tACS performed better on the VM task, while subjects in the ITF-1 group with decreased event-related phase-and power-based connectivity had better learning scores.

Only a few previous studies have focused on theta tACS after effects on behavior^9,26,36–40^. Consistent with most of the previous theta tACS studies, we found a positive after effect of theta tACS on task performance. Furthermore, our study showed that behavioral after effects of theta tACS were prominent in the group that received slower theta stimulation than their theta peak frequency as we hypothesized based on the theta-gamma coupling theory^21,22^. Contrary to our findings, Polania et al.^46^ showed that fronto–parietal theta tACS delivered at anti-phase (180-degree phase difference) decreased working memory performance. However, in contrast to Polania et al., we here focused on after effects (synaptic plasticity) of tACS rather than online effects (entrainment). In this sense, our approach is more similar to Pahor and Jaušovec^40^ who also investigated the effect of offline theta (and gamma) tACS on memory performance and on resting EEG and task-related EEG. Interestingly, Pahor and Jaušovec also reported no significant behavioral after effect of theta fronto-parietal tACS except for a tendency to a small improvement in certain working memory task types^40^. In contrast to all of this previous tACS memory research, however, we here employed an episodic memory task, and we aimed to slow down an individually calibrated theta frequency. This may be particularly relevant when comparing our results to previous findings considering that our behavioral positive after effect exclusively occurred in the ITF-1 tACS stimulation group. Wolinski et al.^28^ studied the online effects of theta tACS as well and showed increased working memory performance with 4 Hz tACS, and decreased performance in the 7 Hz tACS, both as compared to sham tACS. In the current study, even after tACS offset, we observed better behavioral performance in the slowing theta tACS group for VM and learning. Given a large number of behavior and EEG measures in this study, we should consider these findings encouraging but also in need of future replication. At the same time, the VM task was the first task applied immediately after the tACS session, making it the most likely to reveal behavioral tACS after-effects if after-effects were short-lived. Moreover, as expected based on the underlying theoretical models according to which memory capacity can be increased by lengthening theta cycles (i.e., decreasing theta frequency), we found memory improvement specifically and exclusively in the ITF-1 Hz group. On the other hand, a positive effect was seen also in "learning" scores, which were obtained from the applied neuropsychological battery that was administered quite some time after tACS offset. Intervening tasks, including the auditory memory task (applied during the EEG recording) and other neuropsychological tests (digit span-forward, letter-number sequencing test, Rey complex figure) did not show any tACS effects.

The most consistent finding in previous EEG studies of tACS after effects is an increased amplitude in resting state EEG in the stimulation frequency range^32–35,40,50–52^. In accordance with these previous studies, we here also found increased fronto-central power in the targeted frequency range. Intriguingly, this tACs-induced frequency-specific entrainment effects was only observed in the ITF group, i.e., in the condition where tACS was applied based on the individual theta peak frequency. When applying tACS at ITF-1 (slowing), in contrast led to no significant increases in theta power and thus no entrainment effects. At first glance, this might seem in conflict with the cognitive after-effects we seclusively observed for the ITF-1 condition with the reported memory enhancement and associated EEG effects being induced specifically by tACS at 1 Hz slower than ITF. However, as explicitly hypothesized, this is what we expected as tACS consistently has shown to have the most pronounced effect on the EEG power spectrum when it is administered at the individual peak frequency (see also^33,35,48,49^), not when tACS is administered at a flanking frequency deviating from individual peak. In contrast, when it comes to the aim of enhancing memory performance, based on a very different mechanism related to theta-gamma coupling, we should in contrast expect cognitive enhancement in memory capacity specifically in the ITF-1 condition where tACS is designed to shift, i.e., slow, individual theta frequency, but not to affect theta power.

Several studies have shown that tACS applied to change the frequency of oscillations could have an impact on cognitive functions such as working memory and perception^26,28,53^. Vosskuhl et al.^26^ applied tACS at a frequency below ITF and stated that a frequency shift in post-tACS EEG data was not to be expected due to the disappearance of the behavioral after effect immediately following tACS offset; although a frequency shift was not directly assessed in their study. Here, we also did not find a significant “peak frequency” change, despite the revealed behavioral after effects of tACS at ITF-1. This could mean that (1) our behavioral results were false positives, (2) our EEG peak frequency results were false negatives (e.g., underpowered analysis), or (3) the behavioral effects do not require a change in peak frequency. We propose the latter option as our data also indicate that the behavioral after effects arise from a different mechanism than hypothesized, as reflected in the here observed changes in frontoparietal coherence at ITF-1 tACS.

Our EEG results revealed interesting differences between resting-state and task-state (event-related) after effects. We observed increased fronto-central resting theta power in specifically the ITF group as discussed above, but decreased event-related fronto-central theta power in both conditions, tACS at ITF and at ITF-1. Several studies showed that frontal theta increases with increased task demand^54–56^. Therefore, decreased event-related frontal theta after tACS might indicate that tACS at either ITF or ITF-1 showed the same effect, i.e., higher memory performance with lower effort, especially in the ITF-1 group. The observed decreased event-related theta responses for the VM task, not for the AM task, are consistent with this idea of changes in cognitive demand, since behavioral effects of tACS were also observed specifically in the VM task.

A similar opposite pattern between resting-state and event-related data was also observed in coherence analyses when correlating the EEG effects with the behavioral data. Subjects in the ITF-1 group who showed increased resting-state left frontal-parietal theta connectivity after tACS also showed higher scores in the VM task. On the other hand, subjects in the ITF-1 group with decreased event-related phase- and power-based connectivity after tACS showed higher learning scores. This negative correlation seems counter intuitive. However, Berger et al.^59^ showed that dynamic regulation of task difficulty is adjusted through fronto-parietal interaction indicating easier tasks induce fronto-parietal de-coupling between theta and gamma oscillations. In this context, this negative correlation in event-related connectivity may be due to the change in the perceived task difficulty in the ITF-1 tACS group. If so, we should expect an increase in behavioral scores as perceived task difficulty decreases, and this situation manifests itself with a decrease in fronto-parietal theta connectivity. The importance of connectivity between these two regions has been shown in many studies^57–59^, in addition to studies showing the importance of frontal and parietal regions in memory functions. In accordance with these studies, our results showed correlations between the behavioral changes in memory tasks and theta left fronto-parietal connectivity only in the ITF-1 group, and thus again in line with our hypothesis. Even if a direct change or shift in the theta peak frequency following IFT-1 tACS was not shown as the neural mechanism underlying the enhanced memory performance in this condition, it appears that fronto-parietal theta connectivity may be a possible neural substrate of longer-lasting behavioral changes induced by optimal, in our case slowing, tÀCS at ITF-1. Reinhart and Nguyen^9^ also emphasized that tACS- enhanced memory performance may be related to neuroplastic changes in functional connectivity induced by exogenous modulation of theta-gamma characteristics. As such, this may represent a general principle by which EEG-informed fronto-parietal tACS at individually calibrated frequency can exert lasting cognitive changes, namely by systematically stimulating slightly below (or above, depending on neural network and task demand) the individual peak frequency in each participant.

Overall, our findings suggest that left fronto-parietal tACS applied based on the individualized theta frequency may have potentially beneficial impacts on memory and theta brain oscillations, and that these effects of stimulation may continue beyond the duration of the stimulation. However, we should also acknowledge the limitations of this study. We here determined ITF based on a global (whole-head) EEG power spectrum. The literature shows no clear consensus or optimal way on how to estimate ITF: Jaušovec and Jaušovec^39^ found individual alpha frequency in the power spectrum according to the model proposed by Klimesch^45^ and calculated individual theta based on individual alpha frequency. In a few papers, the theta frequency with the strongest coupling to gamma activity in the pre-stimulus state^26^ or in the resting state^8,40^ was accepted as the individual theta of the participant. Reinhart and Nguyen^9^ used PLV synchronization metrics between left temporal and left prefrontal areas during memory maintenance to determine ITF. The enhanced power we found in the resting state specifically in the ITF tACS group is encouraging, suggesting that our approach might have successfully revealed ITF. On the other hand, it is also possible that the theta-range in the global power spectrum is predominantly driven by fronto-central theta oscillations, and that the more optimal procedure to estimate ITF is to only include activity over task-relevant areas (in this case fronto-central). Another limitation of the current study was the ceiling effect in behavioral tasks/tests. Since our participants were healthy, young-adult, and educated, they already achieved very high scores (close to the upper limit) on many of the tests, in the pre-application. Therefore, detection of actual tACS-related score changes in many of the tests may have been difficult to reveal. That is a limitation of our null findings, not our positive results.

All in all, our results provide new insights into oscillatory mechanisms underlying tACS-induced behavioral enhancements that outlast stimulation and may open the door to real-world applications of using NIBS in cognitive rehabilitation and/or neurocognitive enhancement. Reliable after effects of tACS would substantially broaden the range of applications of this technology.

## Methods

### Participants

Although no statistical methods were utilized to calculate sample sizes, our sample sizes are comparable to those published in earlier publications^33,35,52^. The study was designed as a single-blind, between-subject, randomized controlled study. A total of 46 right-handed, educated, healthy young-adult participants were included and randomly assigned to one of three groups: ITF group (N = 15, 13 females, mean years of education (SD): 14.4 (± 1.76), mean age (SD): 20.87 (± 1.99)), ITF-1 group (N = 16, 12 females, mean years of education (SD): 17.63 (± 2.9), mean age (SD): 27.13 (± 5.57)) or sham group (N = 15, 10 females, mean years of education (SD): 17.13 (± 2.2), mean age (SD): 25.27 (± 3.41)). All participants had normal or corrected-to-normal vision and no specified hearing impairment. Participants with symptoms or history of psychiatric or neurological disorders and psychiatric or neurological medication usage were not included in the study. All participants were naive regarding the electrical stimulations, conditions, and tasks.

The study conformed to the principles of the Declaration of Helsinki. Participants provided written informed consent, and there was no compensation for participation as indicated in the written informed consent. The study was approved by the Istanbul Medipol University Ethics Committee (No: 10840098-604.01.01-E.18575).

### Tasks and design

**Figure 4:**
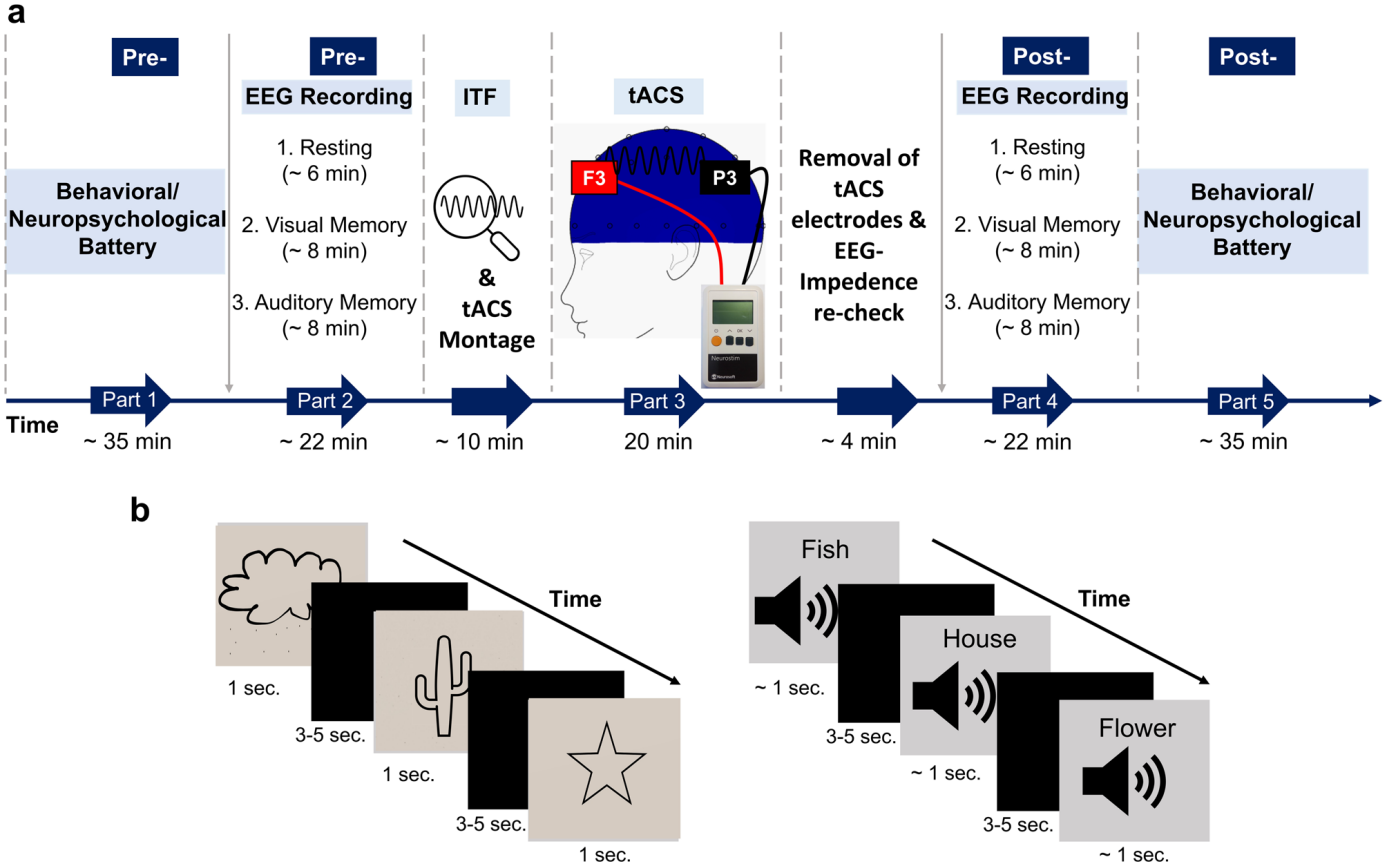
Experimental design and task procedure. (**a**) Design of the experiment. The “part” states each experimental phase. The horizontal dark blue arrow shows the time-course and application order of the parts with durations of them in minutes. The vertical grey arrows show the application order of the numbered measurements stated in the part. (**b**) The representations of the applied visual* (on the left) and auditory (on the right) memory tasks during the EEG recordings. * Given schematic images in Fig. 4b are not actual exemplars from the Boston naming test set; drawn by TA as an example. tACS, transcranial alternating current stimulation; ITF, individual theta frequency; min, minute; sec., second.

The three groups were (1) ITF (tACS applied at individual theta frequency), (2) ITF-1 (tACS applied at ITF11 Hertz), or sham (placebo tACS). Each participant was measured in a single session. The procedure for each session consisted of 5 parts as can be seen in Fig. 4a. First, a battery of behavioral and neuropsychological tests evaluated attention, learning, and memory processes. Second, pre-tACS EEG was recorded in 3 different sections. (1) resting-state EEG was recorded for approximately 6 min (3 min eyes closed, 3 min eyes open), (2) EEG during a visual short term memory task (see below), (3) EEG during an auditory short term memory task. We determined individual theta frequencies (ITFs) of participants from the eyes open resting-state EEG (see “tACS” section for details). Third, we applied tACS/sham for 20 min. No tasks were performed during the tACS, participants were asked to relax and instructed to keep their eyes open. Fourth, we repeated the EEG recordings (‘post-EEG’). And finally, fifth, we repeated the battery of behavioral and neuropsychological tests, identical to the pre-tACS tasks/recordings, albeit with new stimuli (e.g., not repeating memory items from the early visual/auditory memory tasks). To avoid potential order effects, the pre and post versions of the performed tasks and neuropsychological battery were counterbalanced across the participants. Note that the visual memory task was the first task after tACS for all participants.

#### Behavioral and neuropsychological evaluation

Two subtests of the Wechsler Adult Intelligence Scale; digit span forward and letter-number sequencing tests^60^, Oktem verbal memory test (OVMT)^61^, and the Rey complex figure test (RCFT)^62,63^ were administered. The digit span forward test is mainly used for the evaluation of verbal attention while the letter-number sequencing test measures the working memory. OVMT is a commonly used test to measure the verbal memory processes of individuals. The test offers the opportunity to evaluate many components related to verbal memory processes such as verbal short-term memory (max score: 15), long-term memory (max score: 15), and learning (max score: 150). RCFT is used to measure individuals' visual-spatial structuring skills and visual memory processes. The test consists of copying, immediate recall, and delayed recall sections (max score: 36 for all sections).

Additionally, before starting the experiment, participants were asked to rate their belief in the effect of the tACS application (between 0 and 10). Scores close to 0 indicate lower belief, while values close to 10 indicate greater belief in the efficacy of tACS. In this way we measured participants' general opinion/skepticism about tACS efficacy. After the experiment, they were asked whether they thought the tACS they had just received was real or placebo (or “do not know”). Side effects (e.g., itching) were assessed with a 7-item 4-point Likert scale side effects questionnaire. 7 items were as follows: itching, pain, burning, warmth/heat, metallic/iron taste, fatigue, alertness. Each item was rated between 1 and 4, indicating “none” to “strong”. For the statistical group comparison, scores of items were aggregated to a single ‘side effect score’ by summing each response.

#### Task procedure

Our visual and auditory memory tasks were ‘subsequent memory paradigms’ (SMP), prepared and presented using E-prime software (Psychology Software Tools Inc., Pittsburgh, PA). The SMP is used for assessing brain activity generated during episodic learning/encoding that is associated with the later successful recall^11,64,65^. The pictures (black and white drawings) from the Boston Naming Test^66^ were used as the stimuli for the visual memory task. In the visual memory task, during encoding, a selection of object images was shown. In the auditory memory task, we presented audio recordings of items' names taken from the Oktem Verbal Memory Test (a different form relative to the neuropsychological battery), recorded in an isolated room and balanced by decibel and time using Audacity software (Audacity(R)).

In each task, the encoding phase was followed by the retrieval phase. EEG was recorded only during the encoding phase. The stimulus duration of visual images was 1 s, and as much as possible the audio stimulus durations approximated one second as well. The time between stimuli (interstimulus interval) varied randomly between 3 and 5 s (see Fig. 4b). Per task, we presented 25 different stimuli with 3 repetitions, leading to 75 trials in total. The whole sequence of 25 items was presented and then re-randomized for each repetition, but with fixed order of items across participants (pseudo randomization). The visual stimuli were shown in full-screen mode on a 47.5 × 26.8 cm size monitor with a refresh rate of 60 Hz that was placed at a viewing distance of 90 cm. The visual angle for the stimuli measured approximately 19° horizontally and 16° vertically.

Before each task, participants were asked to pay attention to, and remember for later recall, the upcoming set of stimuli (encoding). Immediately after each task, participants were asked to say which items they remembered, and auditory and visual memory free-recall scores were obtained (numbers of items successfully recalled). To ensure that participants understood the tasks, a short practice preceded each memory task presenting 3 different stimuli (non-overlapping with main task stimuli) with 2 repetitions in pseudorandom order. This was only in the ‘pre-tACS’ task blocks where the tasks were introduced to participants for the first time.

### EEG recording

EEG was recorded from Fp1, Fp2, F7 F3, Fz, F4, F8, Ft7, Fc3, Fcz, Fc4, Ft8, Cz, C3, C4, T7, T8, Tp7, Cp3, Cpz, Cp4, Tp8, P3, Pz, P4, P7, P8, O1, Oz and O2 electrodes with “BrainCap with Multitrodes” model cap (EasyCap GmbH, Germany) with 32 electrodes placements based on the international 10–20 system. Two linked electrodes (A1 + A2) were placed to the earlobes as references. The electrooculogram (EOG) was recorded at the medial upper and lateral orbital rim of the left eye. The impedance of electrodes was kept below approximately 10 kΩ. The EEG was amplified by means of a Brain Amp MR plus 32-channel DC system machine (Brain Product GmbH, Germany) with band limits of 0.01–250 Hz and digitized online with a sampling rate of 500 Hz. The participants sat in a dimly lit and shielded room during EEG recordings.

### Data analysis

#### Resting EEG

The preprocessing steps of resting EEG data were performed in Brain Vision Analyzer. The preprocessing steps were as follows; (1) 3 min continuous resting EEG data in eyes-open condition were filtered between 0.1 and 60 Hz, (2) data were segmented to 1-s length epochs, (3) manual artifact rejection was performed over the segmented data.

The preprocessed (cleaned and segmented) data were imported to the FieldTrip Toolbox^67^ for the resting EEG power analysis. Frequency domain power spectrum analysis of resting EEG data at the individual theta frequency range (calculated over the participants’ stimulation frequencies) was performed with FFT by using Hanning tapers. To achieve the 0.1 Hz frequency resolution, epochs were zero-padded. For each participant, power was summed in a 3 Hz window around their tACS stimulation frequency (stimulation frequency + /− 1.5 Hz), and values were exported for statistical analysis. In addition to summed power values, the maximum peak frequencies in the same frequency range were also exported for use in the statistical analysis.

The theta (4–7 Hz) resting coherence analysis between the F3 and P3 electrodes where stimulation electrodes of the tACS were placed were performed over the FFT (Hanning window, zero-padded, 0.1 Hz frequency resolution) in the frequency domain using BVA. Current source density (CSD) was applied on the FFT, before calculating the coherence analysis to attenuate the possible volume conduction effect (CSD parameters: Order of Splines: 4, Maximal Degree of Legendre Polynomials: 10, Default Lambda was used: 1E-05). The mean coherence values between the F3 and P3 electrodes at the specified frequency band (4–7 Hz) were used in the statistical analysis.

#### Event-related EEG

The preprocessing steps and further analyses of the event-related EEG data were performed in BVA. For the event-related EEG data preprocessing steps were as follows; (1) EEG data were downsampled to the 256 Hz, (2) data were filtered between 0.1 and 60 Hz, (3) independent component analysis was applied to remove eye-movement related artifacts, (4) data were segmented into 6-s epochs (3 s before and 3 s after the stimulus) for remembered and forgotten items separately, (5) manual artifact rejection was performed over the segmented data, (6) data were sub-segmented into 2-s epochs (1 s before and 1 s after the stimulus) for the time–frequency analyses.

All event-related analyses were performed for both remembered and forgotten items separately, to compare the after effect of theta tACS on associated event-related EEG activity. Event-related analyses in the time–frequency domain were completed by the Gabor normalized complex Morlet Wavelet Transform (WT) with 3 cycle wavelet widths for theta (4–7 Hz) frequency range. The determined frequency range (4–7 Hz) was subdivided into 60 bins ("frequency steps" parameter was set as "60"), scaled logarithmically (“Logarithmic Steps” option was selected). The WT was calculated in each frequency bin.

In the event-related power analysis, post-stimulus responses were normalized to the pre-stimulus baseline (− 500 to − 300 ms) and converted to decibels (dB). The event-related power was calculated by averaging single trials to which WT was applied to reach total power (evoked + induced power).

Current Source Density (CSD) processing was applied on the segmented data, before the WT to attenuate the possible volume conduction effect for the below-mentioned event-related connectivity analyses (CSD parameters: Order of Splines: 4, Maximal Degree of Legendre Polynomials: 10, Default Lambda was used: 1E-05). After CSD, WT was applied for the event-related connectivity analyses.

The event-related power-based connectivity analysis in the time–frequency domain used in the current study measures the similarity between specified channels across trials. In this method, the sources/channels with high similarity approach values of magnitude 1, whereas low similarity approaches 0. For the event-related power-based based connectivity analysis, WT output values were chosen as “Wavelet Coefficients—Complex Values [µV]”, then magnitude-squared coherence was applied. The event-related power-based connectivity analysis was calculated between F3 and P3 electrodes where the tACS stimulation electrodes were placed on.

The Phase Locking Value (PLV) used in the current study is a phase-based connectivity metric in the time–frequency domain based on the study of Lachaux et al.^68^, and it indicates the phase-lag consistency of two channels by computing the correlation between complex wavelets phases across trials. In this method, if the phase difference between the two channels is constant throughout time, one can conclude that the two channels are well synchronized, causing the PLV to approach 1. If the phase difference is inconsistent, it can be interpreted as the two channels having low synchronization, and the PLV approaches 0. For the PLV analysis, WT output values were chosen as “Wavelet Phase—Complex Values”, then normalized correlation measures were applied, and results were rectified. The PLV was calculated between F3 and P3 electrodes where the tACS stimulation electrodes were placed on.

The sum values of the time window between 50 and 300 ms were divided by the total number of data points in the determined time (50–300 ms) and frequency (4–7 Hz) interval, producing average values (point mean normalization). These mean values were used in the statistical analyses of the event-related EEG.

Outliers were identified and removed from the dataset based on the interquartile range (IQR). Only extreme outliers were removed from the dataset, defined as data points below Q1-3*IQR or above Q3 + 3*IQR. Participant's difference score (post tACS minus pre tACS values) for both EEG measures and behavioral measures was checked for outliers separately for each measure, and if an extreme outlier was detected, they were removed from the dataset before the statistical analyses of the corresponding measure. The removed extreme outliers, if any, were reported in the results section for each analysis.

### TACS

The tACS with maximum 1.5 mA (peak-to-peak) stimulation intensity was applied for 20 min during the rest condition, including 3 s fade-in and 3 s fade-out periods with a Neurostim device (Neurosoft, Ivanovo, Russia). If participants reported that they saw phosphenes or felt uncomfortable with 1.5 mA stimulation, the intensity of stimulation was decreased to the highest stimulation intensity that the participant did not see phosphenes and felt comfortable. Stimulation intensity did not significantly differ between the active tACS groups (ITF: min/max/mean/sd: 1.2/1.5/1.45/0.1, ITF-1: min/max/mean/sd: 0.85/1.5/1.38/0.19). In the sham group, tACS application was also 20 min, at the ITF frequency, but with ineffective 0.2 mA intensity, which has no influence on the neural activity but may deliver a skin sensation similar to active tACS^27,69^. Two pure water soaked-simple sponge electrodes (7 × 5 cm) were placed at the F3 and P3 locations to modulate the left frontoparietal network, globally, which has an important role in memory abilities^6,27,42–45^. Electrode locations were marked using an EEG cap, and stimulation electrodes were placed right under the cap to the marked area after the before-EEG recording and carefully removed after the 20 min stimulation. After removing tACS electrodes, EEG electrodes were re-checked if there were electrodes with increased impedance and corrected accordingly. This procedure took ca. 4 min. The impedance values of the tACS electrodes were kept below 10 kOm. Please note, however, that the conductivity of the pure water-soaked tACS stimulation electrodes may have also been aided by EEG preparation processes we applied earlier. Concretely, prior to tACS, the scalp was already prepared for the pre-tACS EEG recording session with alcohol (%70) (first), followed by the application of EEG preparation gel (abrasive electroconductive gel) for placing the electrodes on the cap. This resulted in an impedance below ~  < 10 kOhm for all EEG electrodes. After the pre-tACS EEG recording, tACS electrodes were placed under the cap on the already cleaned and prepared scalp areas. Although here we have not experienced any difficulties considering electrode impedance or additional side effects when using pure water-soaked stimulation electrode (likely related to the above-mentioned preceding EEG preparation), saline-soaked electrode usage is currently the gold standard for sponge electrodes and we also recommend to rather use saline in any future studies. This may be relevant when aiming to replicate our behavioral tACS findings using our identical tACS procedure as we cannot rule out potential (conductivity) problems with using pure water on the sponges as we did here unless one also follows the exact same EEG preparation methods we applied during the preceding EEG measurements.

The application frequency was set to ITF, which we approximated based on a global power spectrum from eyes open resting-state EEG using Brain Vision Analyzer software (BVA) (BrainVision LLC, Morrisville, North Carolina, United States). The global power spectrum was used to determine the ITF considering that in addition to epicenters in the brain for specific cognitive functions, also a network activity spread over the scalp may have a decisive effect on the cognitive abilities^70^. The steps used to determine ITF were as follows; power spectra were obtained with Fast Fourier Transform (FFT) (Hanning window, zero-padded, 0.1 Hz frequency resolution) for each epoch over the preprocessed data (cleaned and segmented; see “Resting EEG” section for details) and then averaged. ITF was the frequency with maximum power in the theta range (4 to 7 Hz) from all EEG electrodes (excluding Fp1 and Fp2 since they are sensitive to eye movements.). The maximum theta was always chosen as ITF. The ITF did not differ between groups (ITF group: mean/sd: 5.03 ± 1.01 Hz, ITF-1 group: mean/sd: 4.97 ± 1.02 Hz, Sham group: mean/sd: 5 ± 0.99 Hz).

### Statistical analysis

Statistical analysis was performed with IBM SPSS Statistic 22 (IBM Corp., Armonk, N.Y., USA) and Jamovi (The jamovi project, 2021) software^71^.

Chi-Square and Kruskal Wallis tests were used in the statistical analyses of the scores of the tACS-related questionaries.

For the statistical analysis of the behavioral data, Repeated Measures ANOVAs were performed with the 3-by-2 mixed design for each applied test/task. Time (pre-tACS, post-tACS) was the within-subject factor, and Group (ITF, ITF-1, Sham) was the between-subjects factor in the design.

For the statistical analysis of the resting EEG and Event-related power analysis, two sets of analyses were run. In the first set, “location” was added as the within-subjects factor to the ANOVA design to see the possible tACS effect on the main recorded cortical areas. Accordingly, for the resting EEG, mixed-design Repeated Measures ANOVA with the between-subjects factor of group (ITF, ITF-1, Sham) and within-subjects factors of time (pre-tACS, post-tACS), location (7 electrode clusters; Frontal, central, temporal, temporoparietal, parietal-1, parietal-2, occipital), and hemisphere (left, right) was performed. For the event-related power analysis, mixed-design Repeated Measures ANOVA with the between-subjects factor of group (ITF, ITF-1, Sham) and within-subjects factors of time (pre-tACS, post-tACS), location (7 electrode clusters; Frontal, central, temporal, temporoparietal, parietal-1, parietal-2, occipital), hemisphere (left, right), and item encoding (remembered, forgotten) was performed. After the first set of ANOVAs, the factor of location was removed, and analyses were repeated to focus only on the location around the site of tACS stimulation (left frontocentral location).

For the statistical analyses of the EEG connectivity analyses, theta frequency connectivity values between F3 and P3 electrodes were used in the ANOVAs. For the resting EEG coherence analysis, 3-by-2 mixed-design ANOVA was used. Time (pre-tACS, post-tACS) was the within-subjects factor, and group (ITF, ITF-1, Sham) was the between-subjects factor in the design. For the event-related power- and phase-based connectivity analyses, ANOVAs with the between-subjects factor of group (ITF, ITF-1, Sham) and within-subjects factors of time (pre-tACS, post-tACS) and item encoding (remembered, forgotten) were performed.

In order to reveal possible EEG-behavior interaction regarding after effect of theta tACS, correlation analysis and analysis of covariance (ANCOVA) for the repeated measures were conducted.

For the correlation analysis, bivariate linear correlation (Pearson correlation, 2-tailed) analysis was used between behavioral scores and EEG data. As behavioral data, the scores that were significantly affected by tACS according to the results of the ANOVAs were used in the correlation analysis (namely, VM and OVMT total learning scores). As the EEG data, left frontocentral theta values of the event-related power, resting power, and resting maximum peak frequency, and F3-P3 theta connectivity values of the event-related power-and phase-based connectivity, and resting coherence were used. Only the VM task was included in the correlation analysis for the event-related EEG data since there were no shown after effects of tACS on AM task. The difference scores were calculated for both behavioral and EEG data by subtracting the pre-values from the post-values (post minus pre values), and difference scores were used in the correlation analyses.

In order to check whether pre-tACS EEG activity had an effect on the after effect of the tACS on behavior, ANCOVAs for repeated measures were performed for the VM and total learning scores, separately, with the between-subjects factor of the group (ITF, ITF-1, Sham), within-subjects factors of time (pre-tACS, post-tACS), and the “pre-tACS EEG data” as the covariate. Pre-tACS EEG values of the 6 different EEG variables, which were also used in the correlation analysis, were used as the covariates. These covariates were: left frontocentral theta values of the event-related power, resting power, and resting maximum peak frequency, and F3-P3 theta connectivity values of the event-related power- and phase-based connectivity, and resting coherence.

The significance threshold was set at p < 0.05. Greenhouse Geisser corrected p values are reported for the ANOVA and ANCOVA analyses.

## Supplementary Information

Supplementary Information.

## Data Availability

The data that support the findings of this study are available from the corresponding author (Alexander T. Sack; a.sack@maastrichtuniversity.nl) and Tuba Aktürk (t.akturk@maastrichtuniversity.nl) upon reasonable request.

## Code Availability

The data acquisition, processing, and analysis was performed using publicly available software as described in “Methods” section.
