# Supplementary material for: Enhancing memory capacity by experimentally slowing theta frequency oscillations using combined EEG-tACS
Source: Sci Rep. 2022 Aug 20;12:14199. doi: 10.1038/s41598-022-18665-z (PMC9392784; doi:10.1038/s41598-022-18665-z)
Supplement: Supplementary file 1 — Supplementary Information. [file 41598_2022_18665_MOESM1_ESM.pdf]

Enhancing memory capacity by experimentally slowing theta frequency oscillations using combined EEG-tACS

Tuba Aktürk[1, 2, 5], Tom A. de Graaf[2], Bahar Güntekin[4,5], Lütfü Hanoğlu[3, 5], Alexander T. Sack*[2]

[1] Program of Electroneurophysiology, Vocational School, Istanbul Medipol University, Istanbul, Turkey

[2] Section Brain Stimulation and Cognition, Department of Cognitive Neuroscience, Faculty of Psychology and Neuroscience, Maastricht University, Maastricht, Netherlands

[3] Department of Neurology, School of Medicine, Istanbul Medipol University, Istanbul, Turkey

[4] Department of Biophysics, School of Medicine, Istanbul Medipol University, Istanbul, Turkey

[5] Research Institute for Health Sciences and Technologies (SABITA), Istanbul Medipol University, Istanbul, Turkey

***Corresponding author:**

Alexander T. Sack

email: a.sack@maastrichtuniversity.nl

Department of Cognitive Neuroscience, Faculty of Psychology and Neuroscience, Maastricht University, Maastricht, Netherlands

Full Model ANOVA Results:

Resting State EEG Results

In a first set of ANOVA, we included the between-subjects factor of group (ITF, ITF-1, Sham) and within-subjects factor of time (pre-tACS, post-tACS), location (7 electrode clusters; Frontal, central, temporal, temporoparietal, parietal-1, parietal-2, occipital), and hemisphere (left, right). This analysis revealed no after effects of tACS on the resting EEG power (time*group interaction: $F(2, 43) = 0.71$, $p = 0.932$, and $\eta_p^2 = 0.003$) or the maximum peak frequency (time*group interaction: $F(2, 43) = 0.02$, $p = 0.243$, and $\eta_p^2 = 0.064$) in the individualized theta frequency range (no higher-order interactions; all p's>0.05). The location effect was significant in the power analysis ($F(1.72, 73.93) = 9$, $p = 0.001$, and $\eta_p^2 = 0.174$), and the highest theta power values were observed at the frontocentral locations (pairwise comparisons: frontal vs other 5 locations $p<0.003$, central vs temporo-parietal locations $p<0.01$).

Event-Related EEG

The first set of ANOVAs included group (ITF, ITF-1, Sham), time (pre-tACS, post-tACS), location (7 electrode clusters; Frontal, central, temporal, temporoparietal, parietal-1, parietal-2, occipital), hemisphere (left, right), and now additionally item encoding success (remembered, forgotten, post-hoc labeled based on later (un)successful recall). Despite the complexity of this 5-factor model, the ANOVA revealed several interesting effects.

In the auditory memory task ANOVA, there were no after effects of tACS on event-related power for the AM task in the theta band (time*group interaction: $F(2, 29) = 0.76$, $p = 0.474$, and $\eta_p^2 = 0.05$). The location effect was significant ($F(2.6, 77) = 42.53$, $p = 0.0001$, and $\eta_p^2 = 0.595$), with the highest event-related theta power at frontocentral locations (pairwise comparisons: frontal vs. other 5 locations $p<0.007$, central vs. other 5 locations $p<0.001$). The item encoding*hemisphere*group interaction was also significant ($F(2, 29) = 4.9$, $p = 0.015$, and $\eta_p^2 = 0.253$). Accordingly, sham group had higher left theta power during the encoding of remembered items (pairwise comparison, $p=0.018$), with no such difference for the tACS groups (ITF-1; $p=0.350$, ITF; $p=0.440$). Since there were no other indications of any tACS after effects on the auditory memory task, and since we were mostly interested in differences between the ITF-1 and ITF groups, we did not explore these results further.

In the ANOVA on event-related theta-band power during the visual memory task, we found a significant time*location*group interaction (F(4.4, 76.5) = 2.63, p = 0.036, and η_p^2 = 0.131). Following up: decreased event-related frontocentral theta power after tACS was observed in the tACS groups especially in the ITF group (pairwise comparisons: for ITF group, pre-tACS vs. post-tACS for frontal location p=0.014 and central location p=0.035, for ITF-1 group, pre-tACS vs. post-tACS for frontal location p=0.057 and central location p=0.014) compared to the sham group (pairwise comparisons: pre-tACS vs. post-tACS for frontal location p=0.153 and central location p=0.116). The item encoding success*location*hemisphere*group interaction was found significant (F(13.1, 89.4) = 2.55, p = 0.013, and η_p^2 = 0.127): Post hoc analyses on item encoding success conditions showed no difference for location*hemisphere*group interaction (for remembered p=0.439, for forgotten p=0.110). We did not pursue this interaction further because there were no additional signs of tACS after effects on the visual memory task. The location*hemisphere interaction was significant (F(31, 216.8) = 5.01, p = 0.003, and η_p^2 = 0.125): the highest event-related theta power values were observed at the right parieto-occipital locations (for all pairwise comparisons, p<0.001). Accordingly, there were significant main effects of location (F(2.1, 72.1) = 21.94, p = 0.0001, and η_p^2 = 0.385) and hemisphere (F(1, 35) = 4.98, p = 0.032, and η_p^2 = 0.125). Follow-up pairwise comparisons revealed that the parieto-occipital locations had higher theta power compared to the other locations (for all pairwise comparisons, p<0.006), and the right hemisphere had higher theta power compared to the left hemisphere (p=0.032).

Analysis of Covariance

The result of the ANCOVA in which maximum peak frequency of the pre-left frontocentral resting added as a covariate showed only significant covariate effect, suggesting that pre-left frontocentral resting maximum peak frequency significantly related to the after effect of the tACS on the VM scores (F(1, 42) = 8.37, p = 0.006, and η_p^2 = 0.166). There was a negative relation between the pre-resting maximum peak frequency and VM scores: The subjects with lower pre-theta frequency had a higher VM score (for pre-VM scores: r=-0.449, p=0.002, post-VM scores: r=-0.404, p=0.005) (Supplementary Fig. 5). For the remaining ANCOVAs, the behavioral after effect of the tACS on the learning and VM scores in the ITF-1 group remained significant, and the results showed pre-EEG data have no influence on the behavioral after effect of tACS.

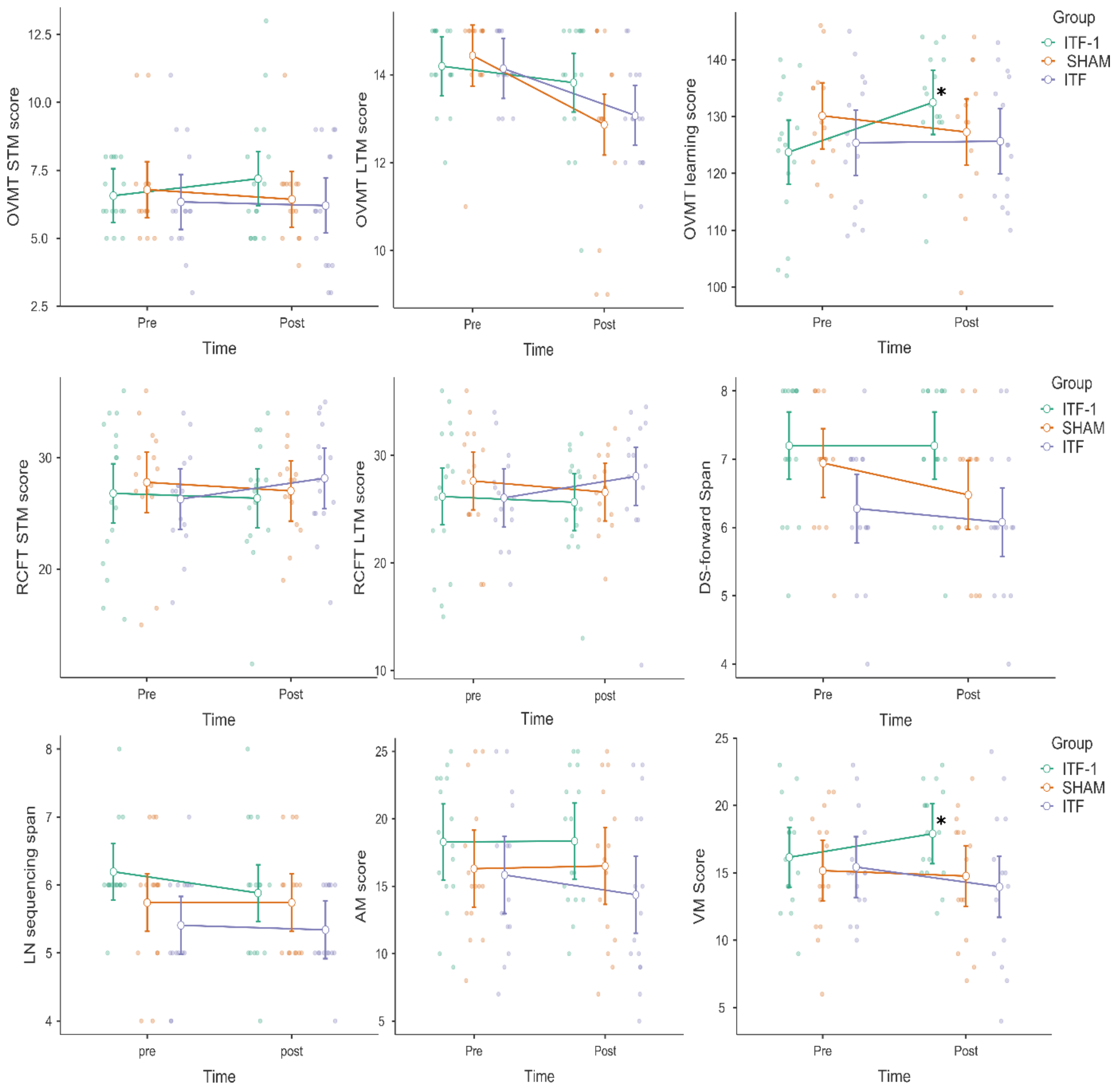

Supplementary Figure 1: The mean values of the behavioral test scores at pre- and post-tACS across the different groups. The ITF-1 group had an increased learning and VM scores after the tACS while there was no time*group interaction for other behavioral measures. OVMT: Oktem verbal memory test, STM: short-term memory, LTM: long-term memory, RCFT: Rey complex figure test, DS: digit span, LN: letter-number, AM: auditory memory, VM: visual memory, tACS: transcranial alternating current stimulation, ITF: individual theta frequency. The vertical bars denote 0.95 confidence intervals. Dots represent the observed scores. Asterisks (*) represent $p<.05$.

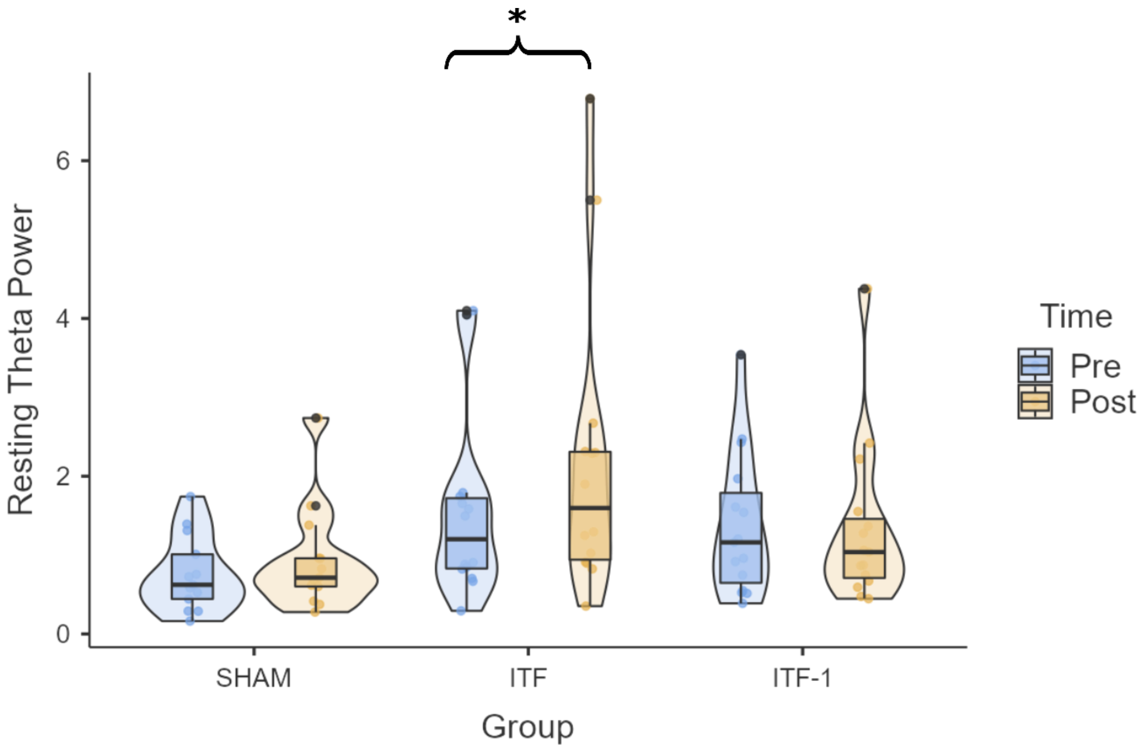

Supplementary Figure 2: The time*group interaction was significant (p = 0.016): ITF group had increased theta power after tACS while there were no tACS effects on the other groups. ITF: individual theta frequency. The vertical bars denote 0.95 confidence intervals. Dots represent the observed scores. Asterisks (*) represents p<.05.

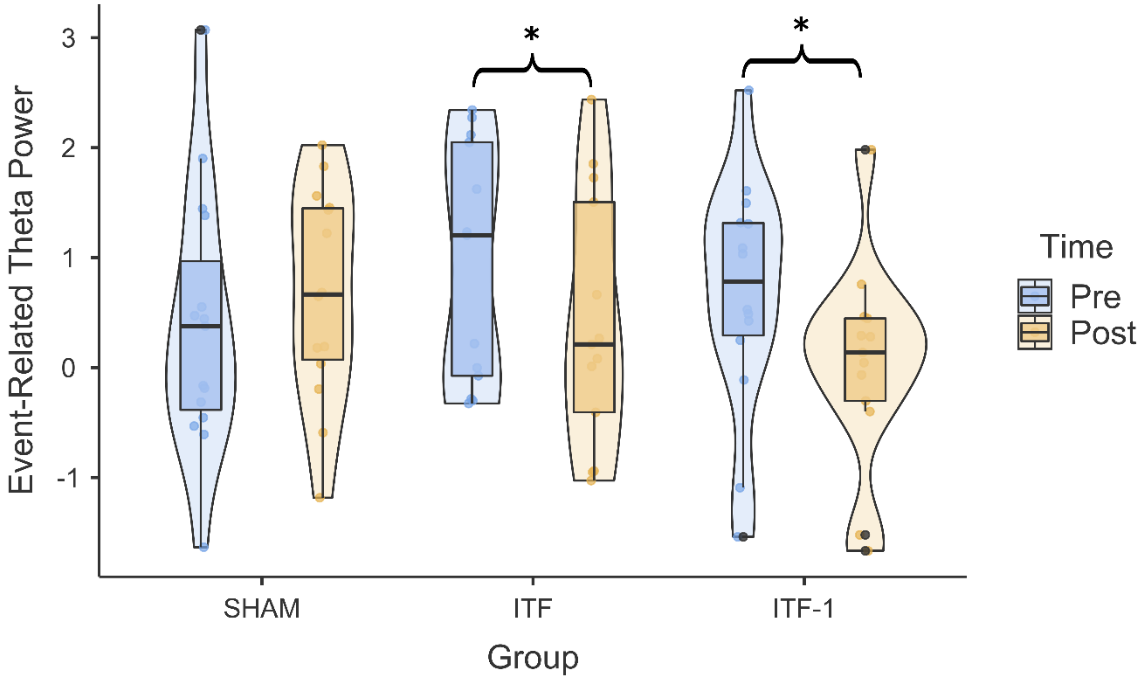

Supplementary Figure 3: The time*group interaction was significant (p = 0.01) for VM task. The tACS groups had decreased event-related theta power after tACS (post) (for ITF-1 p=0.045, for ITF P=0.011) while no such difference was observed in the sham group (p=0.145). ITF: individual theta frequency. The vertical bars denote 0.95 confidence intervals. Dots represent the observed scores. Asterisks (*) represents p<.05.

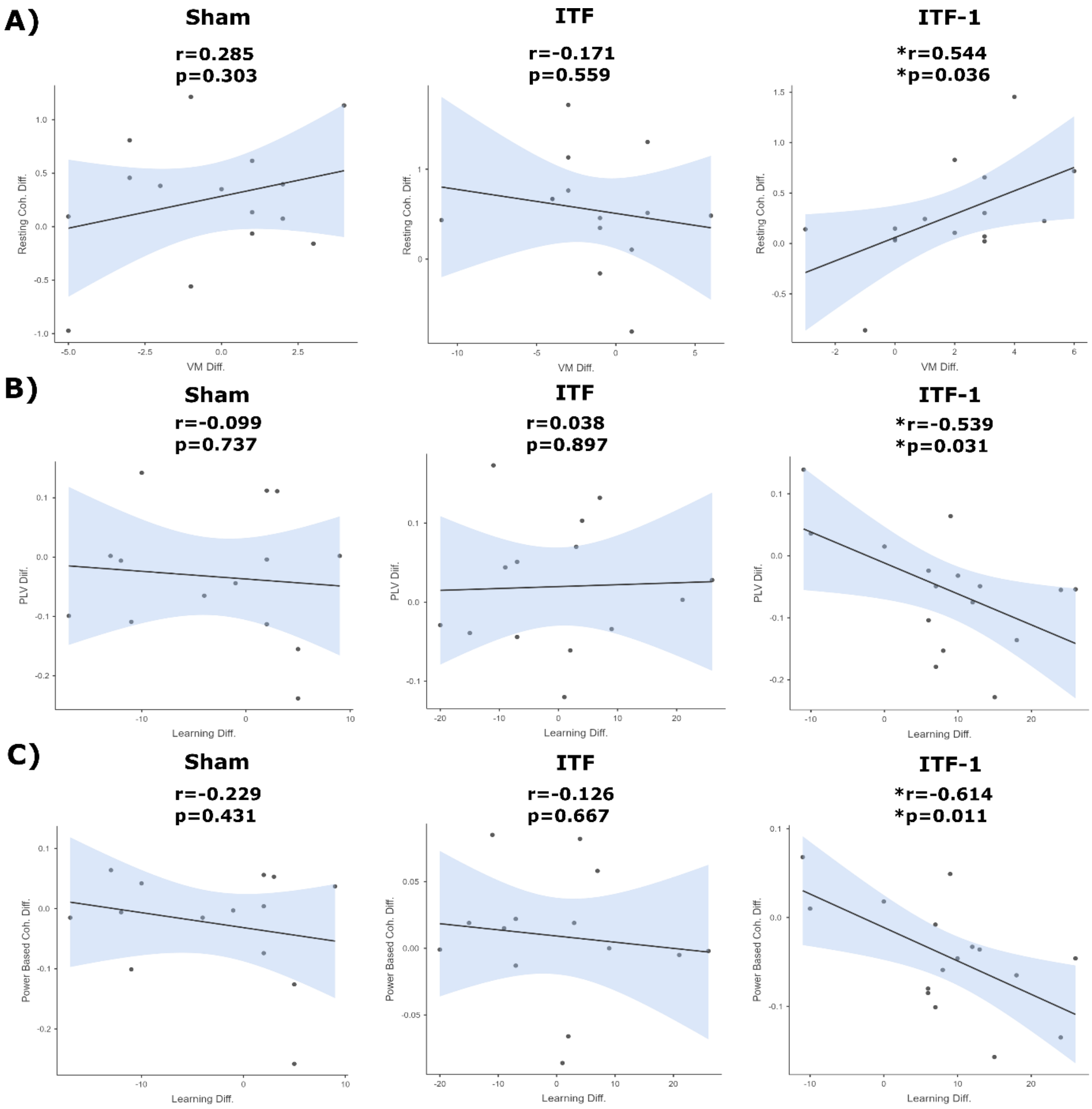

Supplementary Figure 4: A) There was a positive correlation between tACS effects (pre-/post tACS difference score) on left frontal-parietal resting-state theta coherence and the VM task, specifically in the ITF-1 group (r=0.544, p=0.036 ; ITF group p=0.559; Sham group p=0.303) **B)** There was also a negative correlation in specifically the ITF-1 group between tACS effects on specifically learning scores (not VM) and left frontal-parietal PLV (ITF-1 group r=-0.539, p=0.031; ITF group p=0.897; Sham group p=0.737) **C)** We observed a strong negative correlation between tACS effects on left frontal-parietal event-related power-based theta connectivity and learning scores (r=-0.614, p=0.011), again specifically for the ITF-1 group (ITF group p=0.667; Sham group p=0.431). Coh.: coherence, Diff.: difference score, PLV: phase locking value, Max.: maximum, Freq.: frequency, VM: visual memory, ITF: individual theta frequency. The shaded area denotes the standard error. Dots represent the observed scores. Asterisks (*) represents p<.05.

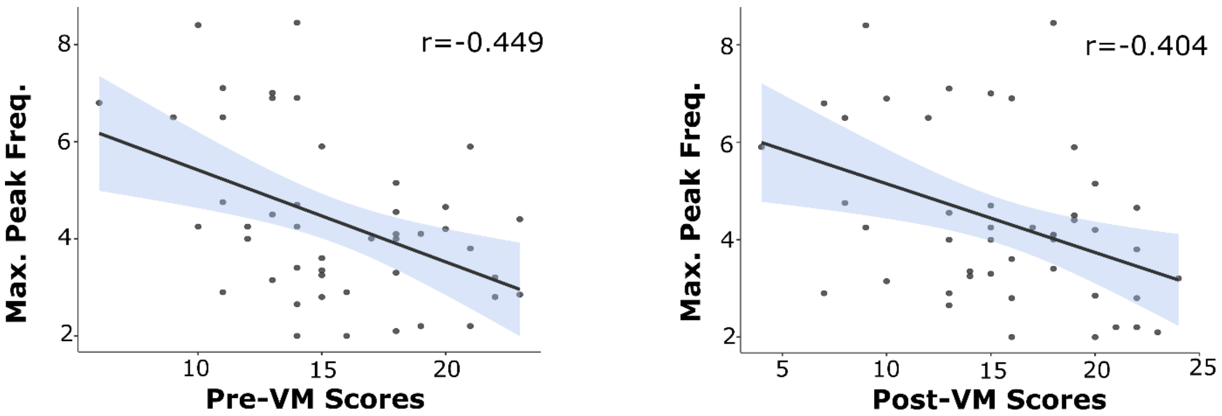

Supplementary Figure 5: The pre-left frontocentral resting maximum peak frequency significantly related to the after effect of the tACS on the VM scores (p = 0.006). There was a negative relation between the pre-resting maximum peak frequency and VM scores: The subjects with lower pre-theta frequency had a higher VM score (for pre-VM scores: r=-0.449, p=0.002, post-VM scores: r=-0.404, p=0.005). Max.: maximum, Freq.: frequency, VM: visual memory. The shaded area denotes the standard error. Dots represent the observed scores.

Supplementary Table 1: Behavioral Results for Tasks

**Tasks (During the EEG recording)**	**ITF-1 (N=16)**		**ITF (N=15)**		**Sham (N=15)**		***P***
	**Pre** Mean SD	**Post** Mean SD	**Pre** Mean SD	**Post** Mean SD	**Pre** Mean SD	**Post** Mean SD	
Auditory Memory: Free-recall Scores	18,2 ± 5	18,3 ± 5	15,8 ± 5,7	14,3 ± 6,4	16,3 ± 5,2	16,5 ± 5,8	0.400
Visual Memory: Free-recall Scores	16.1 ± 3.9	17.9 ± 3.4	15.4 ± 3.9	13.9 ± 5.6	15.1 ± 4.5	14.7 ± 4.7	0.016*
	pre < post		-		-		0.026

SD: standard deviation. ITF: individual theta frequency. P values belong to the results of Group X Time interaction. Asterisks (*) represents p<.05.

Supplementary Table 2: Behavioral Results for Neuropsychological Tests

**Neuropsychological Tests**	**ITF-1 (N=16)**		**ITF (N=15)**		**Sham (N=15)**		***P***
	**Pre** Mean SD	**Post** Mean SD	**Pre** Mean SD	**Post** Mean SD	**Pre** Mean SD	**Post** Mean SD	
**Oktem Verbal Memory Test:**							
Short-term Memory	6.6 ± 1.2	7.2 ± 2.4	6.3 ± 2.1	6.2 ± 2.3	6.7 ± 1.9	6.4 ± 1.6	0.472
Long-term Memory	14.2 ± 0.9	13.8 ± 1.5	14.1 ± 0.7	13.1 ± 1.2	14.2 ± 1.1	12.9 ± 2.1	0.065
Total Learning	124 ± 12.2	133 ± 9.8	125 ± 11.8	126 ± 10.9	129 ± 9.7	127 ± 12.2	0.011*
	pre < post		-		-		0.002
**Rey Complex Figure Test:**							
Short-term Memory	26.8 ± 6.6	26.4 ± 5.4	26.3 ± 4.5	28.2 ± 5.1	27.8 ± 5.6	27 ± 4	0.539
Long-term Memory	26.2 ± 6.8	25.7 ± 4.8	26.1 ± 4.4	25.7 ± 4.8	28.1 ± 6	26.6 ± 3.7	0.458
**Digit Span Forward**	7.2 ± 1	7.2 ± 0.9	6.3 ± 1	6.1 ± 1.1	6.9 ± 0.9	6.5 ± 1	0.581
**Letter-Number Sequencing Test**	6.2 ± 0.7	5.9 ± 1	5.4 ± 0.8	5.3 ± 0.6	5.7 ± 1	5.7 ± 0.8	0.626

SD: standard deviation. ITF: individual theta frequency. P values belong to the results of Group X Time interaction. Asterisks (*) represents p<.05.
